# Comparison of Indirect Immunofluorescence and Enzyme Immunoassay for the Detection of Antinuclear Antibodies

**DOI:** 10.7759/cureus.31049

**Published:** 2022-11-03

**Authors:** Mohammad J Khalifah, Omar Almansouri, Syed Sameer Aga, Ammar A Aljefri, Abdulaziz Almalki, Naser Alhmdan, Wael Al-Mazain, Khalid Alsalmi, Abdulfattah Alamri

**Affiliations:** 1 Rheumatology, King Saud Bin Abdulaziz University for Health Sciences College of Medicine, Jeddah, SAU; 2 Medicine and Surgery, King Saud Bin Abdulaziz University for Health Sciences (KSAU-HS), Jeddah, SAU; 3 Biochemistry, King Saud Bin Abdulaziz University for Health Sciences College of Medicine, Jeddah, SAU; 4 College of Medicine, King Saud Bin Abdulaziz University for Health Sciences, Jeddah, SAU; 5 College of Medicine, King Saud Bin Abdulaziz University for Health Sciences, King Abdullah International Medical Research Centre, King Abdulaziz Medical City, National Guard Health Affairs, Jeddah, SAU; 6 Pathology and Laboratory Medicine, ‏King Saud Bin Abdulaziz University for Health Sciences, King Abdullah International Medical Research Centre, King Abdulaziz Medical City, National Guard Health Affairs, Jeddah, SAU

**Keywords:** saudi arabia, rheumatology, autoimmune disease, enzyme-linked immunosorbent assay, elisa, indirect immunofluorescence assay, ifa, systemic lupus erythematosus, sle

## Abstract

Objective: The detection of autoantibodies directed toward nuclear antigens is one of the main criteria for the diagnosis of systemic lupus erythematosus (SLE), for which the most commonly used techniques are the enzyme immunoassay and immunofluorescence assay (IFA). However, the sensitivity and specificity of these tests vary between different techniques. Thus, in this study, we aimed to determine the superior method for detecting antinuclear antibodies (ANAs) and compare the accuracy of tests ordered by rheumatologists versus non-rheumatologists.

Materials and methods: We compared the sensitivity and specificity of the two assays in 149 patients from a non-selected population, who were sent to the immunology laboratory of King Abdulaziz Medical City, Jeddah from 2018 to 2019.

Results: The sensitivity and specificity of the indirect IFA were 77.78 % and 58.65%, respectively. The positive and negative predictive values of IFA for SLE were 44.87% and 85.92%, respectively. The sensitivity and specificity of the enzyme-linked immunosorbent assay (ELISA) were 77.78% and 80.77%, respectively. The negative and positive predictive values of ELISA for SLE were 63.64% and 89.36%, respectively. The highest number of false-positive IFA tests was requested by family physicians and the lowest was requested by rheumatologists.

Conclusion: Our data show that IFA has a higher negative predictive value, while ELISA has a higher positive predictive value. The positive predictive value of the test can be improved by pre-selecting patients by specialist rheumatologists.

## Introduction

Antinuclear antibodies (ANAs) are commonly used in the diagnosis of several autoimmune diseases [1], including systemic lupus erythematosus (SLE), Sjorgen’s syndrome, systemic sclerosis (SS), and dermatomyositis [2]. According to the European League Against Rheumatology (EULAR) and the American College of Rheumatology (ACR), the detection of ANA is considered a key part of the immunologic domain of the new joint diagnostic criteria for SLE [3]. SLE is a chronic inflammatory autoimmune disorder that affects many organs [4]. Moreover, due to the progressive nature of the disease, it can lead to high mortality and morbidity if left undiagnosed and untreated [4].

Globally, the incidence of SLE has been reported to be higher in the Americas, Asia, and Australia, with the highest incidence in North America (23.2/100,000 person-years, 95% CI: 22.4, 24.0), and a lower incidence in European countries, with the lowest incidence in Africa (0.3/100,000 person-years) and Ukraine (0.3/100,000 person-years, 95% CI: 0.0, 1.5) [5]. The prevalence of SLE was reported to be the highest in the USA at 0.24% [mk4] (95% CI: 130, 3352) [5]. Unfortunately, not many studies have been carried out on the prevalence of SLE in Saudi Arabia. One study conducted in the central region of Saudi Arabia in 2002 reported a 0.019% estimated prevalence of SLE in Saudi Arabia [6]. In addition, according to the Saudi General Authority for Statistics, majority of the population of Saudi Arabia is between the ages of 15 and 44 - the age group reported to have the highest incidence of SLE [7,8]. Therefore, with the presence of such a large portion of the population in this high-risk group, it is of great importance to be able to accurately diagnose SLE.

Currently, the indirect immunofluorescence assay (IFA) is the gold standard for the detection of ANA in patients with SLE [1]. At present, ANA detection can be performed by IFA, enzyme-linked immunosorbent assay (ELISA), and other techniques. Some doubt has been raised by recent studies regarding which method of ANA detection is actually superior [9,10]. For instance, a recent study that compared the false negatives of different IFA kits (4.9%-22.3%) to the false negatives of ELISA (11.7%) in the detection of ANA yielded similar results for both tests [9]. In a similar study, ELISA was shown to have greater sensitivity (90%-97%) in comparison with IFA (80%) for the detection of ANA [10]. Due to the invention of several methods for the detection of ANA in patients with SLE, most prominently ELISA, it has become of the utmost importance to start an investigation with the aim of determining which method excels on the basis of specificity and sensitivity [1,11]. Hence, in this study, we have attempted to compare the two most widely used methods for the detection of ANA (ELISA and IFA) on the basis of specificity and sensitivity in a [DR10] population who were acquired as a part of a routine procedure.

## Materials and methods

Study design and setting

This study was carried out in The National Guards Health Affairs Hospital at King Abdulaziz Medical City, Jeddah during a period of one year (2018-2019). Ethical approval from the Institutional Review Board of King Abdulaziz International Medical Research Center (KAIMRC) (IRB number SP19/205/J) was acquired prior to the commencement of the study process. The study was conducted in accordance with the declaration of Helsinki.

A retrospective chart review was conducted for samples ordered by different specialties for the detection of ANA. A total of 149 consecutive patient samples received by the immunology laboratory were included in the study (duplicate samples were excluded). A single sample for each patient was tested using the QUANTA Lite ELISA (INOVA Diagnostics, CA, USA) and the NOVA Lite Hep-2 IgG kit (INOVA Diagnostics, CA, USA), following the manufacturer’s procedure. Sera for IFA testing was tested at a screening dilution of 1:80. The IFA was independently read by two senior technologists who were blinded to the clinical diagnosis. Discrepant results were verified by the consultant scientist. Positive sera by either of the procedures were tested for specific antibodies. The detection of specific antibodies and final clinical diagnosis by a specialist rheumatologist were considered the reference results.

 Study sample

The sample size was calculated by Raosoft based on the sensitivity of IFA (97%), with a confidence interval of 95%, a margin of error of 5%, and a test population size of 20,000. The minimum number of necessary samples was determined to be n=45 (5). We included all patients who underwent ANA testing during a one-year period in our center. The actual sample size collected was n=149.

Data collection process

ELISA and IFA sensitivity and specificity for ANA detection were calculated based on records from the conducted ELISA and IFA tests and compared against clinical diagnostic criteria, both of which were collected by medical students from laboratory files and the BestCare system, which is a patients’ electronic record system used at KAMC. The data collection sheet was designed by the investigation team and contained variables including age; sex; specialty requesting the tests; SLE diagnosis; treatment received for SLE; clinical symptoms including fever, rash, joint pain, and other symptoms; and x-ray or MRI findings.

Data analysis

Data were entered and organized into an excel sheet. After that, the data were coded and analyzed through SPSS V.23, which was used as the chosen statistical software for this study. Variables were measured with ANOVA and represented by bar graphs. A P-value < 0.05 was set as to be significant. The calculation of specificity, sensitivity, positive predictive value, and negative predictive value was done using the following formulas: specificity = [true negatives/healthy population]×100, sensitivity = [true positives/diseased population]×100, positive predictive value = [true positives/total positive test results]×100, negative predictive value = [true negatives/total negative test results]×100. Tables 1, 2 show the different variables used in the calculations.

**Table 1:** Variables collected and used for calculating the sensitivity, specificity, positive predictive value, and negative predictive value of IFA. P-value > 0.05

	Diagnosis for SLE		
	Positive	Negative	Total test result
IFA positive	35 (44.9%)	43 (55.1%)	78
IFA negative	10 (14.1%)	61 (85.9%)	71
Total Diagnosis	45	104	149

 

**Table 2:** Variables collected and used for calculating the sensitivity, specificity, positive predictive value, and negative predictive value of ELISA. P-value > 0.05

	Diagnosis for SLE		
	Positive	Negative	Total test result
ELISA positive	35 (63.6%)	20 (36.4%)	55
ELISA negative	10 (10.6%)	84 (89.4%)	94
Total Diagnosis	45	104	149

## Results

Demographics and requesting specialties

The sample population consisted of 98 female and 51 male patients, aged 2-90 years old with a mean of 44.43 ± 19.06 years. The final diagnosis was SLE in 45 patients, nine patients had other autoimmune disorders, while no autoimmune disorders were found in 95 patients. Seventy-six of the ANA tests were requested by internal medicine departments other than rheumatology, 35 of them were requested by rheumatology, 24 by family medicine, nine by the general surgery department, and five were requested by the hematology department. The most common reason for ordering an ANA test was joint pain (85%), followed by skin rash (60%). 

Outcome data 

IFA was found to have equal sensitivity to ELISA in detecting SLE (77.78%). However, IFA had a lower specificity (58.65%) compared to ELISA (80.77%). In the detection of SLE, IFA had a lower positive predictive value, but a higher negative predictive value compared to ELISA; further details can be found in Table 3. When evaluating the positive results of either test, the combined sensitivity of both procedures was 91% and the combined specificity was 49.44%. The positive and negative predictive values were 47.67% and 91.67%, respectively. Four out of 45 SLE patients were negative for both procedures. When other autoimmune diseases that are known to be due to nuclear antigens, such as Sjogren’s syndrome, are included (54 patients), IFA had a higher sensitivity than ELISA (81.4% vs 77.78%). ELISA, however, had a higher specificity than IFA (78.72% vs 64.22%). The positive and negative predictive values when considering other-autoimmune diseases were both higher in ELISA than in IFA; further details are illustrated in Table 4.

**Table 3:** Sensitivity, specificity, positive and negative predictive values for the diagnosis of SLE alone

	IFA	ELISA
Sensitivity	77.78%	77.78%
Specificity	58.65%	80.77%
Positive Predictive Value	44.87%	89.36%
Negative Predictive Value	85.92%	63.64%

**Table 4:** Sensitivity, specificity, positive and negative predictive values for the diagnosis of any autoimmune diseases

	IFA	ELISA
Sensitivity	81.4%	77.78%
Specificity	64.22%	78.72%
Positive Predictive Value	56.41%	67.74%
Negative Predictive Value	85.92%	86.05 %

False-positive results distribution

**Figure 1:**
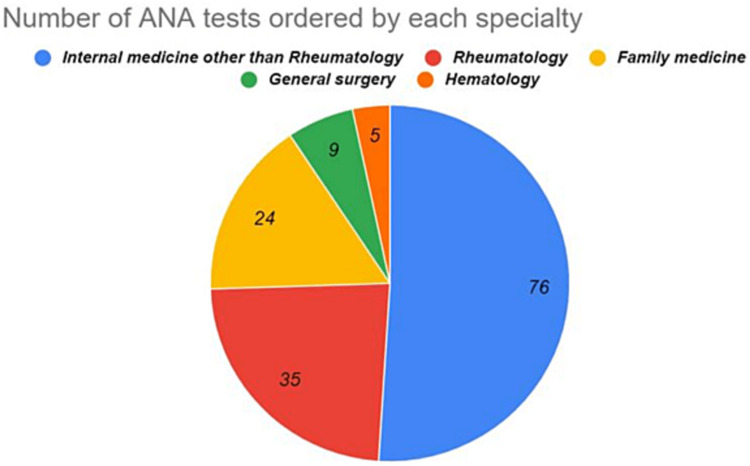
Representation of the number of ANA tests and the corresponding specialty

**Figure 2:**
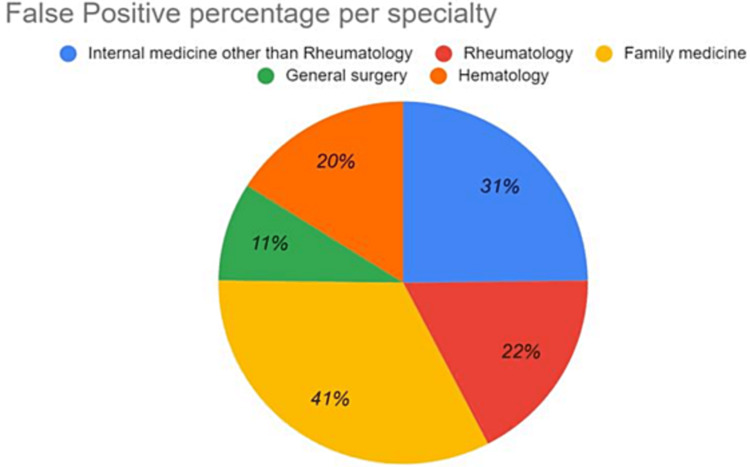
Representation of the false positive tests and the corresponding requesting specialty

False-positive results were obtained from 31 females and 15 males, which was similar to our patient population. Figure 1 represents the number of ANA tests requested by each specialty. The proportion of the results that were falsely positive can be seen in Figure 2. The highest percentage of false-positive results was requested by primary health care (n=10; 41%) followed by internal medicine (n=24; 31%), Rheumatology (n=8; 22%), and other departments (n=2; 22%). However, these differences were not statistically significant (P-value = 0.44).

 

## Discussion

The clinical diagnosis of systemic autoimmune diseases is a challenging task since the clinical signs and symptoms of these diseases overlap with many other conditions. Therefore, the detection of autoantibodies in nuclear components is fundamental for the diagnosis of systemic autoimmune diseases [12]. The choice of algorithm and the procedure of testing make a great contribution to the validity of the results. Our hospital is a tertiary health care facility with six affiliated primary care centers and a secondary care section within the facility. ANA testing can be requested by any physician. In this study, we evaluated the sensitivity, specificity, negative predictive value, and positive predictive value of ANA detection using IFA and ELISA in 148 consecutive non-selected patients attending our center.

The first observation in our study is the high number of positive non-rheumatologic patients, 41%. This high false-positive ANA in the normal population has been previously observed in several studies [13-15]. However, the very high percentage of false-positive ANA in our patient population may be due to the fact that many of our patients are high-risk patients with other diseases [13]. A second possible explanation is that our population is endemic to Dengue fever, which may cause positive ANA results [16]. Regarding the sensitivity of IFA in SLE patients, our results (77.78 %) were slightly lower than previously reported studies. Sharmin et al. reported a sensitivity of 86.5% and Bentow et al. reported a sensitivity of 81.5%. However, our results were higher than the reported 59% sensitivity of Karumanchi and Oommen [17-19].

In our study, the sensitivity of IFA and ELISA were similar (77.78%). Previous studies showed that the sensitivity of the two tests was close to each other [18-21]. In contrast, the specificity of the two tests was different, whereas ELISA was more specific than IFA. This observation was also previously reported using different ELISA and IFA kits [18-21]. These variables were reflected in the positive and negative predictive values of the two procedures, where IFA showed a higher negative predictive value but a lower positive predictive value for SLE compared to ELISA.

Regarding the requesting department and its effect on the sensitivity and specificity of the test, we noticed that the majority of the tests were ordered by non-rheumatologists, and the most common reason for requesting an ANA test was joint pain. Requests made by primary health care physicians were associated with a higher number of false-positive ANA tests compared to other specialties. The observation that false results of ANA were associated with a lack of pre-selection of patients and the requisition by non-rheumatologists has been noted in the literature [13]. These observations led international expert groups to emphasize the need to restrict ANA testing to patients who were pre-evaluated by specialist rheumatologists, and to avoid the use of ANA testing as a screening test in low-risk populations [12,22,23].

In this study, we included all patients who underwent ANA testing at our center over a one-year period. A higher sample size would have given this study more power. We did not include ethnicity or consanguinity in our analysis, as these sets of data are not routinely collected at our center.

## Conclusions

In conclusion, our data showed that IFA is more sensitive but less specific than ELISA for the detection of ANA in patients suspected of SLE. In addition, IFA had a higher sensitivity for the detection of ANA in patients suspected of any autoimmune disease, but it had a lower specificity than ELISA for the same patient population.

While differences were demonstrated between the rate of false positive results based on the ordering specialty, these differences were not statistically significant. It follows, though, that the positive predictive value of the tests can be improved by pre-selecting patients after diligent consultation by specialist rheumatologists.
